# Design and evaluation of a Mobile-Based decision support system to enhance lung transplant candidate assessment and management: knowledge translation integrated with clinical workflow

**DOI:** 10.1186/s12911-023-02249-6

**Published:** 2023-08-01

**Authors:** Hamidreza Abtahi, Leila Shahmoradi, Shahideh Amini, Marsa Gholamzadeh

**Affiliations:** 1 Pulmonary and Critical Care Medicine Department, Thoracic Research Center, Imam Khomeini Hospital Complex, Tehran University of Medical Sciences, Tehran, Iran; 2 Health Information Management and Medical Informatics Department, School of Allied Medical Sciences, Tehran University of Medical Sciences, Tehran, Iran; 3 Halal Research Center of IRI, FDA, Tehran, Iran; 4 Clinical Pharmacy Department, Faculty of Pharmacy, Tehran University of Medical Sciences, Tehran, Iran; 5 Ph.D. in Medical Informatics, Health Information Management and Medical informatics Department, School of Allied Medical Sciences, Tehran University of Medical Sciences, Tehran, Iran

**Keywords:** Clinical decision support system, Lung transplantation, Clinical protocols, Expert system, M-health

## Abstract

**Background:**

Accurate and timely decision-making in lung transplantation (LTx) programs is critical. The main objective of this study was to develop a mobile-based evidence-based clinical decision support system (CDSS) to enhance the management of lung transplant candidates.

**Method:**

An iterative participatory software development process was employed to develop the ImamLTx CDSS. This study was accomplished in three phases. First, required data and standard clinical workflow were identified according to the literature review and expert consensus. Second, a rule-based knowledge-based CDSS application was developed. In the third phase, this CDSS was evaluated. The evaluation was done using the standard Post-Study System Usability Questionnaire (PSSUQ 18.3) and ten usability heuristics factors for user interface design.

**Results:**

According to expert consensus, fifty-five data items were identified as essential data sets using the Content Validity Ratio (CVR) formula. By integrating information flow in clinical practices with clinical protocols, more than 450 rules and 500 knowledge statements were extracted. This CDSS provides clinical decision support on an Android platform regarding inclusion and exclusion referral criteria, optimum transplant time based on the type of lung disease, findings of initial assessment, and the overall evaluation of lung transplant candidates. Evaluation results showed high usability ratings due to the fact provided accuracy and sensitivity of this lung transplant CDSS with the information quality domain receiving the highest score (6.305 from 7).

**Conclusion:**

Through a stepwise approach, the ImamLTx CDSS was developed to provide LTx programs with timely patient data access via a mobile platform. Our results suggest integration with existing workflow to support clinical decision-making and provide patient-specific recommendations.

**Supplementary Information:**

The online version contains supplementary material available at 10.1186/s12911-023-02249-6.

## Introduction

Lung transplantation (LTx) is an established treatment option for patients with end-stage lung disease [1, 2]. Though this surgery can save the lives of many patients with severe pulmonary disease, the success of this operation depends on long-term patient follow-up and the accurate identification of candidates [3–8]. While clinical protocols and criteria for patient management exist, lung transplant centers use different for addressing the different challenges of patient management [9]. Furthermore, the high complexity and availability of such protocols present an additional barrier to accurate utilization, especially for junior healthcare providers [10]. Consequently, LTx centers often face low adherence or improper utilization of clinical protocols at the point of care for transplant candidates’ management [11].

One of the strategies for translating clinical protocols into practice is the use of computerized clinical decision support systems (CDSSs) [12]. Even though designing such information infrastructures remains a complicated and time-consuming task in clinical medicine [13, 14]. Potentially, a CDSS can provide clinicians with evidence-based knowledge in real-time [15–17]. In fact, the literature suggests that physicians demonstrate a positive interest in using mobile health applications for decision support [18]. Despite the modest benefit of improving healthcare provider clinical support in complicated clinical applications [19], there is a paucity of developed and applied CDSSs for the assessment and management of lung transplant candidates [20].

The lung transplant assessment process usually consists of a series of decisions that integrate a large amount of patient data with standard clinical protocols. Accurate assessment could be affected by multiple factors, such as poorly collected data, the high volume of information, and barriers to effective communication among transplant team members [21]. Hence, one suggested strategy is the design of knowledge-based CDSS that integrates with a dynamic clinical environment. Though such systems offer recommendations based on the best clinical evidence, the final decision is made by a physician [22, 23]. Because the accurate identification and management of a suitable transplant candidate based on standard clinical protocols can have a positive impact on the outcome of lung transplantation, the main objective of this study is to design and evaluate a clinical decision support system for the assessment, management, and follow-up of LTx candidates based on the established clinical protocols to improve clinical decision-making.

## Setting

This study was carried out in collaboration with the Lung Transplant Center of Imam Khomeini Hospital Complex Tehran. To achieve balanced usage of information and expertise from several disciplines, a panel of experts was formed in the first phase of this project. This expert panel was compromised of lung transplant clinicians with research experience who met on regular basis during the development and evaluation phases of the lung transplant CDSS.

## Material and methods

**Fig. 1:**
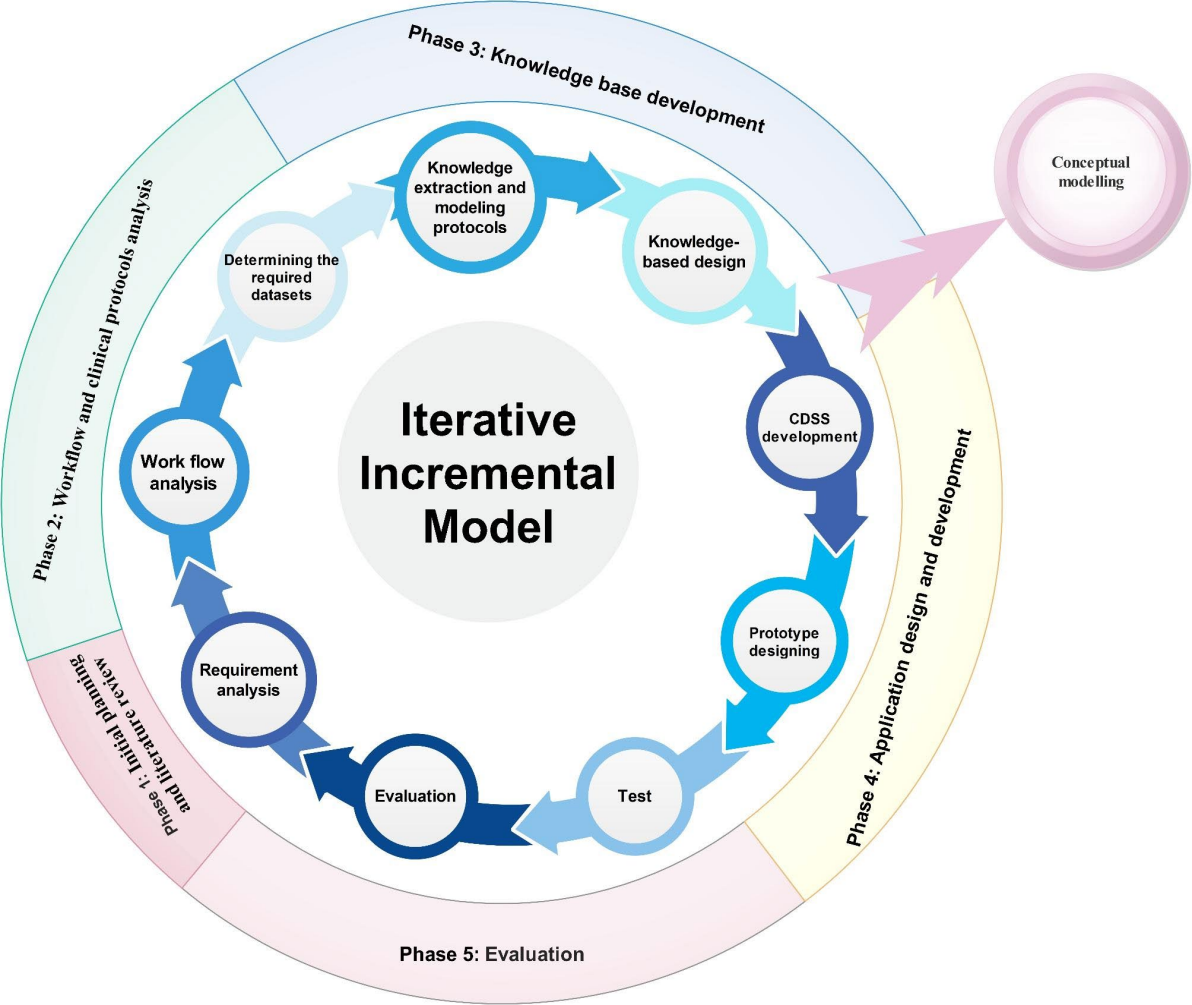
The methodology model of our study

An iterative participatory software development process was employed to develop our CDSS which is entitled ImamLTx CDSS. The different phases of the design and development methodology are shown in Fig. 1.

### Phase1: Initial planning and literature review

To determine the research gaps and requirements regarding medical informatics application in lung transplantation studies, a systematic review in combination with expert consensus was carried out by the authors [20]. Simultaneously, similar existing systems in other countries were identified and investigated through this survey in terms of source of knowledge, features, and performance.

### Phase 2: Workflow and clinical protocols analysis

The current lung transplant candidate management workflow was examined by direct observation and verbal interviews with transplant care team members. Transplant team members were asked about the main processes that occur in lung transplant candidates’ management and their preferences, ideas, and recommendations. Additionally, the interaction between physicians and patients was recorded. The observer (MG) spent approximately four hours per week in the outpatient clinic to examine patient management processes. Subsequently, the optimized workflow of candidate management was designed by eliminating irrelevant and additional processes. Established clinical pathways were analyzed in detail in collaboration with LTx experts to determine key decision points. The extracted knowledge of protocols in the knowledge modeling phase was combined with the results of the enhanced workflow.

### Determining the required minimum datasets and technical structure

To determine the required data items, a descriptive and cross-sectional method was employed after analyzing the workflow. After conducting a literature review, paper-based medical records of our lung transplant clinic were also reviewed to extract a draft of potential datasets. Third, a three-point Likert scale questionnaire was formed based on the preliminary data elements to gather experts’ views that were collected using the data extraction form containing 63 items in eight domains. This questionnaire was distributed among lung transplant experts who had experience working in academic centers of lung transplantation. The panel of experts in this stage consisted of eight experienced lung transplant specialists.

The content validity of our questionnaire was calculated using the Content Validity Ratio (CVR) formula in which n_e_ is the number of experts stated data item is essential and N is the total number of experts. The critical value for CVR is determined based on the Lawshe Table  [24].

$$CVR = \frac{{{n_e} - \left( {{\raise0.7ex\hbox{$N$} \!\mathord{\left/{\vphantom {N 2}}\right.\kern-\nulldelimiterspace}\!\lower0.7ex\hbox{$2$}}} \right)}}{{{\raise0.7ex\hbox{$N$} \!\mathord{\left/{\vphantom {N 2}}\right.\kern-\nulldelimiterspace}\!\lower0.7ex\hbox{$2$}}}}$$

The reliability was determined using Cronbach’s alpha [25]. Cronbach’s alpha is a tool for measuring reliability or internal consistency. The descriptive analysis of the results from the questionnaire-based survey was performed using SPSS version 25.

**Phase 3: Knowledge base development**.

***Knowledge extraction and modeling***.

High-value content was extracted from established protocols and clinical pathways. This step was performed by holding several focus group discussions with a medical informatician and lung transplant experts until reaching an agreement. Upon interviews, the main decision points of the LTx process were recognized by integrating extracted knowledge with enhanced workflow. Ultimately, the clinical pathways were classified according to these decision points by LTx experts in our research team for knowledge modeling.

***Knowledge-based design and development***.

Subsequently, a knowledge management team (KM) completed the translation of the extracted clinical pathways, instructions, and protocols to decision models or decision trees by casting knowledge in the form of IF–THEN–ELSE rules [26]. The KM team consisted of LTx transplant physicians, a medical informatics specialist who had expertise in translating clinical protocols to machine-understandable format, and two clinicians.

Developing the knowledge base of the system was done using the decision tree classifier and ontology suggested by Ertuğrul et al. [27]. Decision rules and actions were encoded based on predefined ontologies using Web Ontology Language (OWL) and Semantic Web Rule Language (SWRL) language. The rules were written using the ROWL plugin by the KM team. Embedded clinical protocols in KB in the form of If-Then-Else rules and SWRL include a set of recommendations that simulate the clinical decision-making procedure employed by clinicians to diagnose, treat, follow up, or manage the LTx candidates.

Next, actionable recommendations, alerts, and patient-specific diagnoses or treatment plans were produced by combining patient data into embedded knowledge through a reasoning mechanism done by an inference engine. Forward-chaining or straight-forward approach was applied in the inference engine of our CDSS. The forward chain of the inference engine explores the facts, conditions, and derivatives before inferring the result and makes a decision based on the available data. In other words, this process works as a transmission chain, from the initial state to the (final) goal (decision) [28]. The inference engine combines the embedded knowledge in its knowledge base with input data (symptoms) to provide the best therapeutic or diagnostic recommendation to the user.

**Phase 4: Application design and development**.

The features and characteristics of the final system were obtained through frequent meetings with the team of experts and requirement analysis in an iterative manner. In these meetings, the group of experts discussed what information should be provided in each section, the number of main sections in the program menu, how to display checklists and assessment plans, data entry, main menu, how the program interacts with the user, and how to reduce errors when using the program.

***CDSS and Prototype development***.

**Fig. 2:**
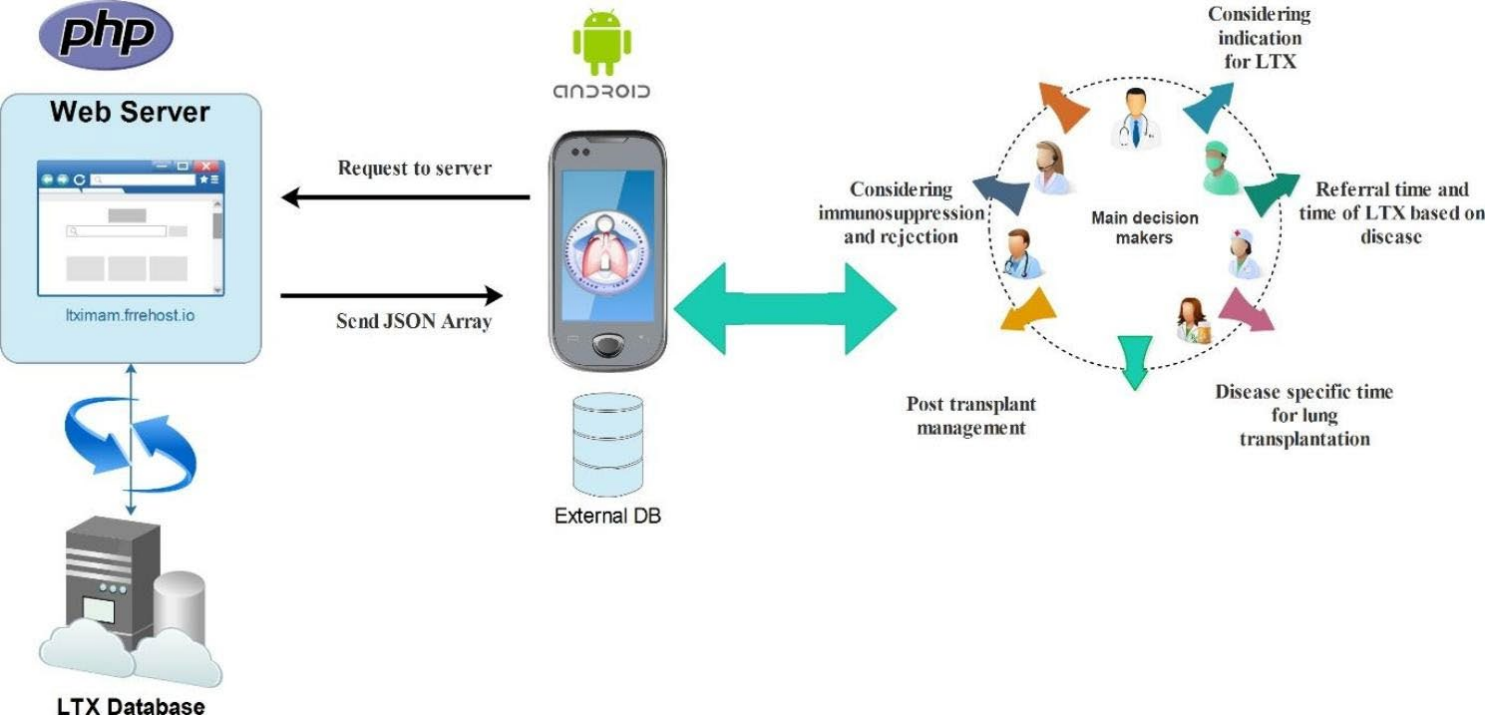
The architecture of the ImamLTx CDSS.

The ImamLTx CDSS was developed as a web-based mobile application. All of the functional procedures and clinical reasoning processes (from patient referral to candidate LTx phase) were converted into an expert system using the Android Java language. Android platform was selected for our CDSS because the majority of studies regarding developing smartphone-based systems for health and medical care used the Android operating system [29]. In the front end, PHP scripting language with MySQL database engine was employed for data storage. The web interface was developed using CCS3 (cascading style sheets), HTML 5, and PHP language. The application relied on a JSON API web service to transfer data. This application used JSON language to send data to the web server (Fig. 2). Evaluation of user inputs and decisions are made based on the decision models embedded in the program. [insert Fig. 2 here]

The initial prototype included information about candidates for a lung transplant, such as demographics, initial evaluation results of available medical records, and decisions regarding treatment plans and referral to the LTx committee. The quality and accuracy of the prototype were evaluated in iterative steps and modified based on the expert’s feedback. Based on the opinions of the evaluators, the prototype was modified many times to reach the usability evaluation stage.

### Phase 5: Evaluation

#### Test and evaluation

Three stages of the evaluation were accomplished to ensure that the ImamLTx CDSS meets all requirements. Firstly, the accuracy and performance of our CDSS were evaluated with real patient data to determine any syntax or functional errors. Targeted sampling was done in selecting patients to cover different lung conditions/diseases. To diminish the bias, a performance evaluation of our application was done by two general physicians who were not LTx team members. Clinical judgment by expert lung transplant physicians in areas of diagnosis, treatment plan, or/and recommendations was set as the golden standard to determine the accuracy and outcome recommendations of the ImamLTx CDSS. This investigation was done by manual review and comparison of these patients’ profiles and the CDSS performance. Consequently, if the physician’s assessment outcomes of the paper-based medical record for a particular patient were identical to the CDSS’s recommendations for the same patient based on the entered information, it was considered as really true (True positive). The following formula was used to calculate the accuracy of the ImamLTx CDSS.

$$Accuracy = \frac{{TP + TN}}{{TP + TN + FP + FN}}$$

Secondly, the application was offered to be used by a limited number of clinicians using the Heuristic Evaluation Checklist [30]. This checklist is composed of the 10 Nielsen heuristics factors [31] which assess applicability, ease of use, and facility feedback after seven days of usage in clinical practices. Clinician evaluators recorded their suggestions.

Thirdly, usability evaluation was completed through a standard seven-Likert-based Post-Study System Usability Questionnaire (PSSUQ) [32]. The PSSUQ was introduced by International Business Machines Corporation (IBM) to collect users’ opinions regarding usability with three main sections, namely system usefulness, information quality, and interface quality with questions ranging from 0 (strongly disagree) to 7 (strongly agree). Participants were recruited using a convenience sampling method from medical staff who are experienced in lung transplantation in Iran. They were asked to complete an online usability questionnaire after one week of working with the system independently. Once the volunteers agreed to participate in the study, the final version of the program and electronic form of the PSSUQ questionnaire was sent to them. Written consent was obtained from participants at the beginning of the survey.

## Results

### Expert panel

The expert panel of this study consisted of ten experts whose demographic characteristics are outlined in Table 1. [insert Table 1 here]

**Table 1:** The demographic characteristics of the expert panel

	Data	Frequency
Specialty	Pulmonologist	4(40%)
	Pharmacotherapist	1(10%)
	Nurse	1(10%)
	Specialist in health information management	1(10%)
	Medical informatics specialist	1(10%)
	General Physician	2(20%)
Age	30–40	2(20%)
	41–50	7(70%)
	> 50	1(10%)
Experience	< 5 years	2(20%)
	5–10 years	5(50%)
	> 10 years	3(30%)

### The results of two phases of initial planning and workflow analysis

The literature review showed that lung transplant systems usually were developed based on the protocols utilized in each center according to the needs and facilities of the transplant team. The established protocols in this center were considered the main source of our knowledge. Accordingly, LTx workflow analysis revealed existing gaps in the processes of selection of lung transplant candidates.

**Fig. 3:**
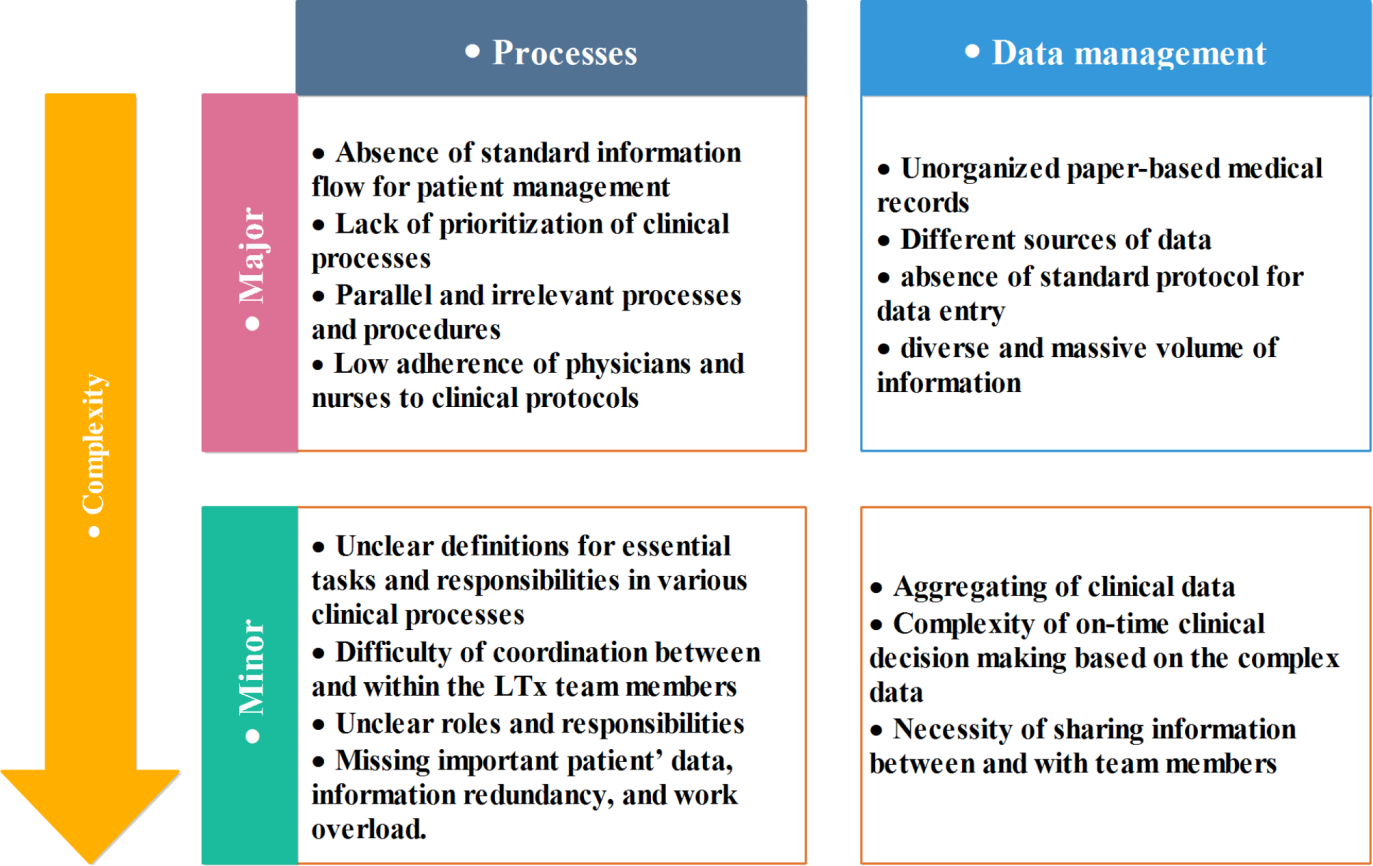
The identified problems and challenges

More than 96 h of direct observations and face-to-face interviews were conducted across the clinic to understand the information flow in lung transplantation thoroughly. By completing the stages of need and workflow analysis, key roles (n = 10), main processes, and sub-processes (n = 57) were identified. Then, all of the recognized gaps and requirements were organized into major or minor categories which are shown in Fig. 3. [insert Fig. 3 here]

**Fig. 4:**
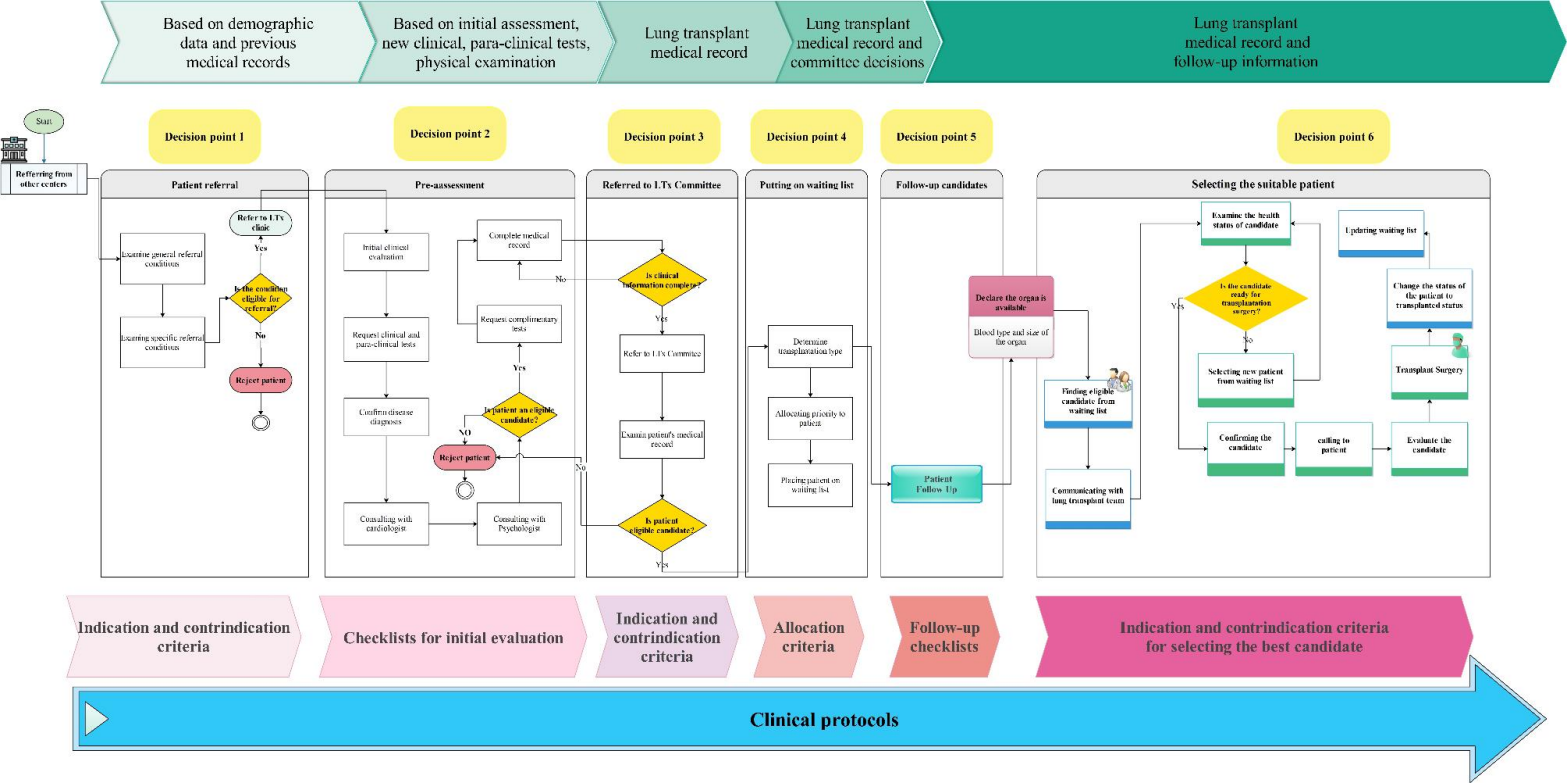
The six-point decision model and information flow of candidate management

During the clinical protocol modeling phase, the content of available clinical protocols was divided into four areas of management: (1) inclusion and exclusion criteria for patient referral, (2) optimum transplant time based on disease diagnosis, (3) initial assessment of the referred patient, and (4) evaluation processes of lung transplant candidates. By merging the results of clinical workflow analysis with the extracted clinical knowledge, a six-point decision model was conceived that focuses on the optimal evaluation of transplant candidates (Fig. 4). [insert Fig. 4 here]. In this six-point model, each of the decision points represented the sequential aggregation of patient data and the updated status of a transplant candidate.

In addition to the above, this prototype model was reviewed and discussed with the lung transplant team for feedback and identification of key functional requirements. Table 2 shows a list of those functional and non-functional requirements that allowed further modifications. The requirements approved by the LTx team member were considered the main features of the final system. [insert Table 2 here]

**Table 2:** Functional and non-functional requirements

Functional requirements	o Possibility of recording clinical and demographic data in a structured way and creating a medical record for each patient o Preparing automatic reports on the latest status of patients based on different groups o Creating a smart form for collecting patient data for each patient based on her/his health status o Immediately alert physicians regarding the patient’s need for more assessment or if not an eligible candidate o Categorizing patient information and test results and making related suggestions for better clinical decision-making in different circumstances o Proposing the most suitable transplant candidate based on blood type matching and required information o Automatically create a transplant day form for the selected patient based on the latest recorded information and send it to transplant team members to save time o Creating automatic statistical reports
Non-functional requirements	o Online, fast, and easy access to patient data from anywhere at any time (High Availability) o Stepwise physician guidance on how to evaluate patients with different conditions and diseases by answering different questions (Performance) o Sending an automatic message to the patient to be selected as a transplant candidate for a routine office visit (Reliability) o Need to create an environment such as a chat room to discuss and coordinate between team members (Usability) o Easy navigation between different forms and sections (Performance)

#### Determining the required minimum data sets

After getting an expert opinion about the datasets and completing of expert review, datasets with a mean score of 3.6 and above were confirmed. The demographic characteristics of the experts who participated in this phase are described in Table 3.

**Fig. 5:**
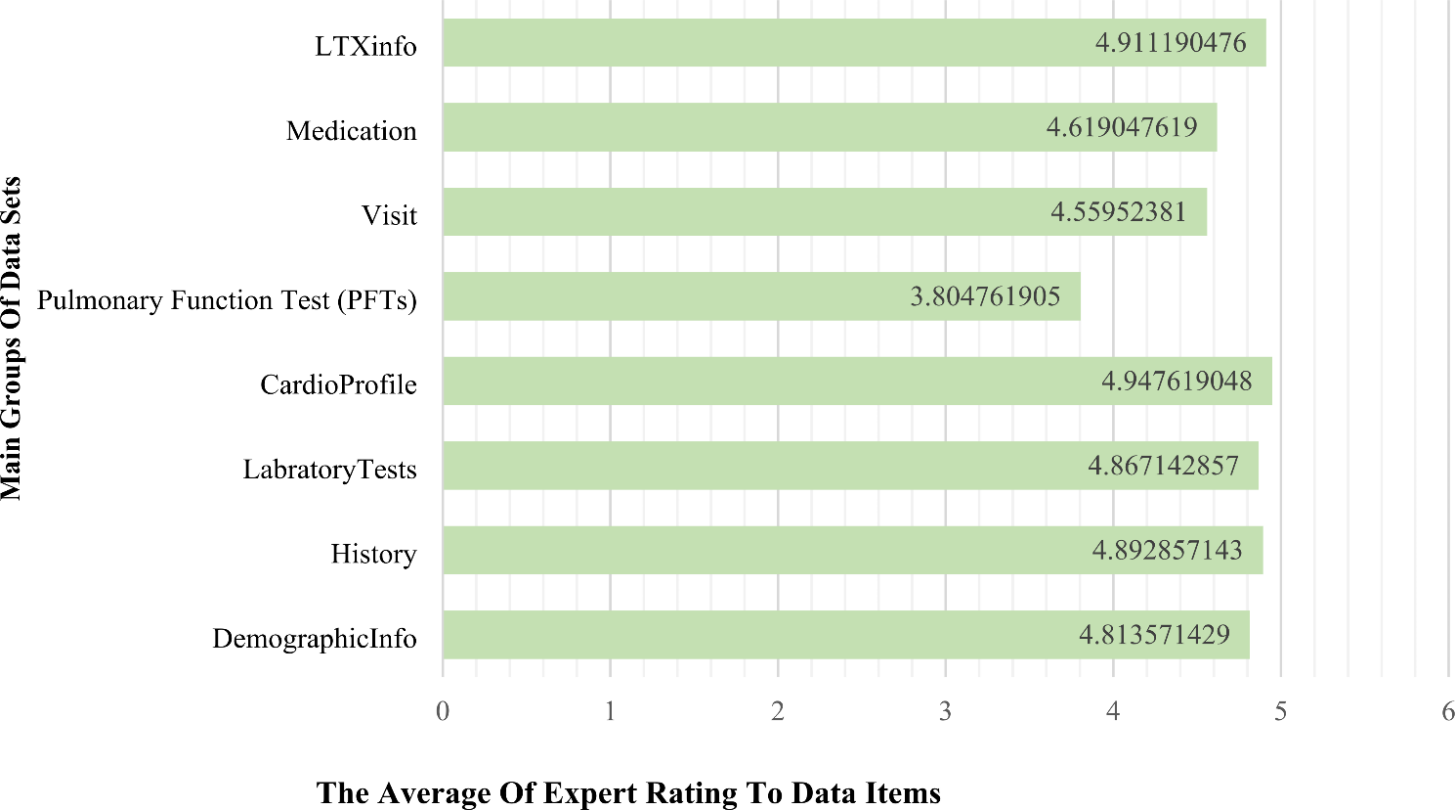
Summary of approved data sets by the experts in eight categories

Datasets with a mean score of less than 3.5 were discarded. Here, fifty-five data items were identified as essential out of the original sixty-three potential data sets collected using the survey method. Figure 5 shows the average score for eight categories of data items. The results of the statistical analysis and CVR values are shown in Table A-1, Appendix A. Additionally, Cronbach’s alpha correlation coefficient was evaluated for the whole questionnaire as 0.936 which is considered excellent [33, 34].

**Table 3:** Demographic characteristics of the experts who identified the minimum dataset

Sex	Frequency	Percentage
Male	5	63%
Female	3	38%
**Expertise**		
More than 20 years	2	25%
11–20 years	3	38%
6–10 years	2	25%
Less than 5 years	1	13%
**Specialty**		
Pulmonologist	2	25%
Cardiologist	1	13%
Internal medicine	1	13%
Pharmacologist	1	13%
Thoracic surgeon		
Medical doctor	2	25%
**Age group-y**		
20–40	0	0%
41–50	3	38%
51–60	3	38%
> 61	2	25%

### Knowledge modeling and knowledge-based development

More than 95 statements were extracted from clinical protocols. Then, those statements were converted into 20 decision trees (DTs) within the different phases of the LTx model. The decision trees were organized and organized into six categories within the routine workflow and clinical protocol. As we mentioned, Fig. 4 shows the six categories which include: major indication and contraindications for patient referral (13 decision trees), criteria for patient initial assessment (two decision trees), placing the patient on the waiting list (one decision tree l), patient follow-up (three decision trees), and selecting the candidate when the donor is available (one decision tree). Each category contains a series of decision models to simulate the clinical decision-making process by LTx experts at each stage of decision-making. Some decision models for candidate assessment that were formulated based on specific lung diseases are illustrated in Fig A-1, Appendix A. All decision trees within the six categories were translated into the “If-then” rules by a medical informatics specialist. Some of the extracted rules regarding the inpatient referral section are represented in Table 4. In total, more than 450 rules and 500 knowledge statements were extracted and converted to logical rules. The extracted rules were confirmed by the multidisciplinary team members. [insert Table 4 here]

**Table 4:** Developed rules for referral investigation

		Example of extracted rules:
The initial level of decision	General criteria for patient referral	**Age**: if (age > = 65 and age > = 14) then “patient has age criteria” and age = true **ESLD**:1- Is the patient in the end stage of lung disease? -> ESLD = True 2- Does the patient have less than a 50% chance of surviving? -> Probability = True 3- Does the patient have advanced lung disease despite the optimal treatment? -> Advanced = True
The second level of patient evaluation for referral to the LTx center	Determine and investigate the NYHA class	Has the patient had no symptoms in his physical activity with no limitations? -> if yes, NYHA = Class I Has the patient had mild symptoms only in normal activity? -> if yes, NYHA = Class II Has the patient symptoms during daily activity, and is asymptomatic only during rest? -> if yes, NYHA = Class III Has the patient severe limitation symptoms even in rest? -> if yes, NYHA = Class IV
The third level of patient evaluation for referral to the LTx center	Confirm disease diagnosis	Are the tests and history of disease verified diagnosis? -> DX = True Does his/her disease include the permitted disease in our center? -> Disease = True
The fourth level of evaluation is related to a patient referral to the ltx center	Investigate the criteria based on the disease	**If we assume that the patient has COPD**: If (FEV1 > 35%) -> FEV1 = True If the patient has a history of severe dyspnea? -> Dyspnea = True If the patient has a history of recurrent hospitalization? -> Hosp = True If (NYHA class = 3 OR NYHA class = 4) -> NYHA = True If (FEV1 = True AND Dyspnea = True AND Hosp = True AND NYHA = True) -> **The patient has the criteria of referral**

The extracted rules were mostly devoted to decision-making (40%) around indications and contraindications. Referral to the LTx committee had the least number of rules (6%). However, in the candidate follow-up section, 20% of the rules were repeated in the initial assessment section.

The knowledge base in the ImamLTx CDSS consisted of general knowledge applicable to all referred patients and a rule-based knowledge base specific to each patient. The patient data stored in the system database constitute the fact data sets that match the left sides of the If-Then-Else rules to determine fitting rules to execute. The Inference Engine (IE) or reasoner of the system associates the most appropriate rules with the fact data (most relevant rules about patient gathered data) to generate the best recommendations to clinicians. All processes were completed in an iterative process under expert supervision to refine and revise to achieve validated knowledge statements.

### Design and development of the CDSS

**Fig. 6:**
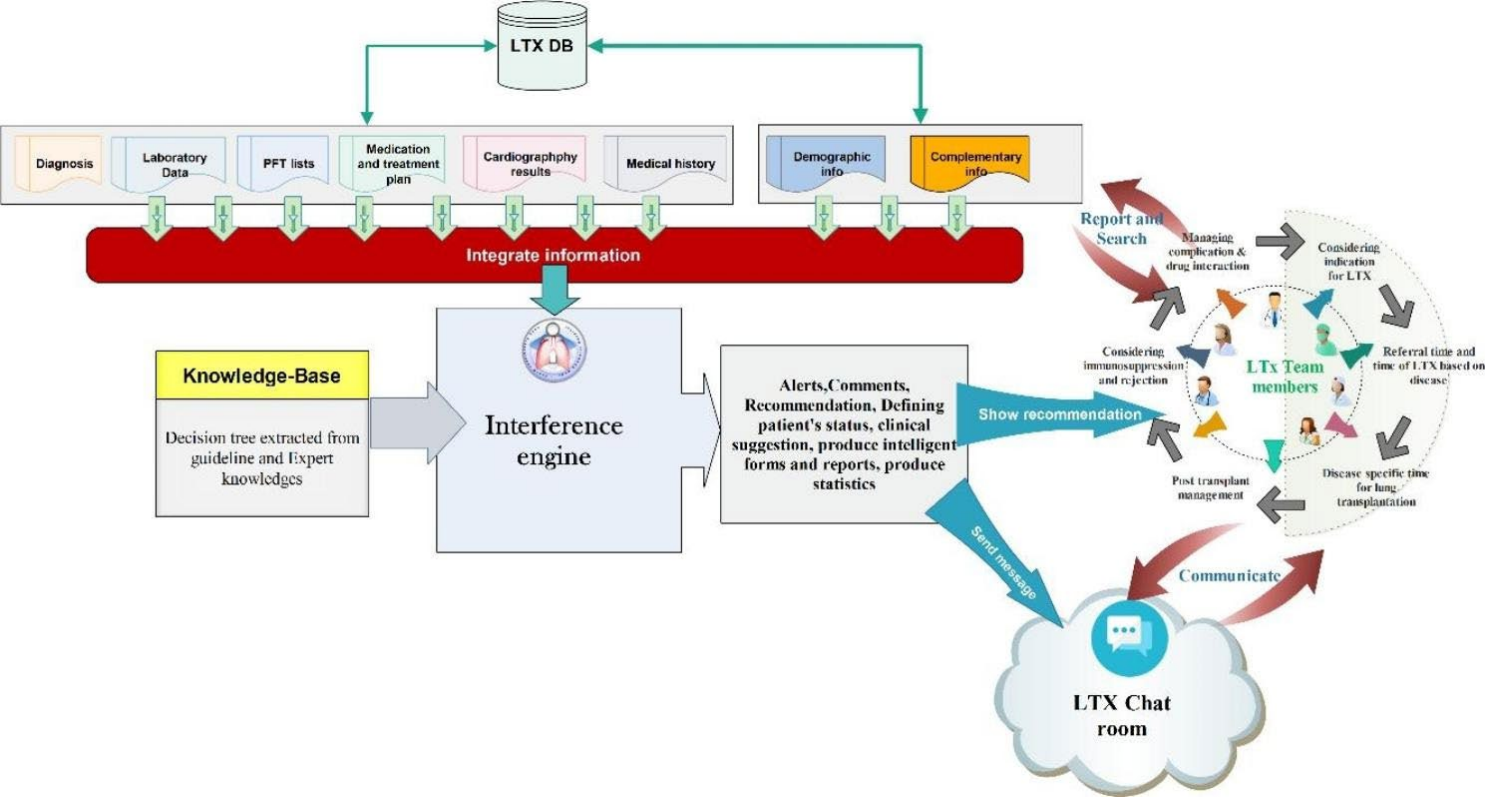
The conceptual model of the CDSS for lung transplantation management

After consultation with clinical experts and an extensive analysis of clinical protocols, a conceptual model was devised to develop the ImamLTx CDSS. Although the proposed model could be modified depending on the specific needs of each lung transplant center, this framework can be suggested as the general framework for managing LTx candidates. This framework is shown in detail in Fig. 6. [insert Fig. 6 here]

**Fig. 7:**
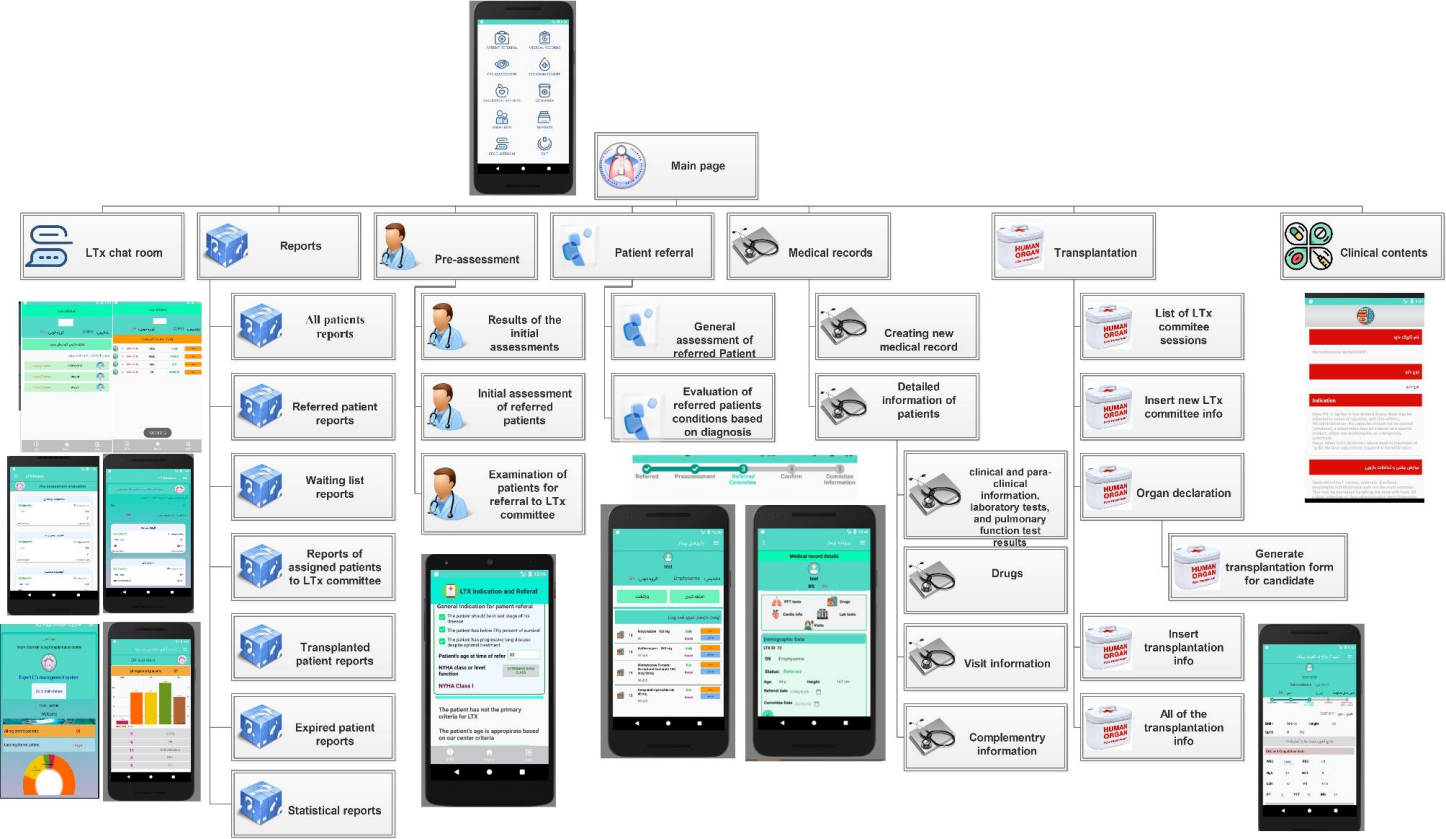
The LTxImam application

Following the completion of the need assessment, determining functional/non-functional requirements, and modeling of the knowledge for decision making, a web-based application (ImamLTx CDSS) was developed for Android Studio which is an integrated development environment that utilizes Java language for coding. Software analysis of the ImamLTx CDSS identified the need for twenty-five parts to cover the seven core tasks of decision-making. Figure 7 shows the different parts of the ImamLTx CDSS supporting the assessment of lung transplant candidates. [insert Fig. 7 here]

Typical usage of LTxImam in clinical practice for the assessment of a lung transplant candidate is illustrated in Fig A-2 and Fig A-3, Appendix A. First, after an initial log-in by a user, the new patient is assessed by meeting the criteria of referral to the LTx based on the established decision tree. If the referral conditions are met, then the system will guide the user to create a medical record for the patient. The patient data is saved in the MySQL database. Users can retrieve and complete patient records through a simple search.

In all patient evaluation processes, the most appropriate suggestions, warnings, and criteria that should be considered are displayed to the user. At the same time, after completing the patient information and being validated by the physician, the patient’s status automatically changes, for example, the patient’s status changes from the referral status to the waiting list after being approved by the LTx committee.

Patients’ follow-up information on the waiting list can also be recorded through this application. The system displays recommendations to the physician if further testing or consultation is required. On transplantation day, by adding the information about the blood group of donors, the list of patients who are eligible for the transplantation and their information is shown to the user. When a lung transplant candidate is selected, all test results and clinical data are generated automatically. After the selected patient is confirmed by a physician, the initial information about the patient will be sent to the chat room, and the team members are informed. Ultimately, surgical information can be recorded into the system to change the patient’s condition to a transplant patient automatically. By applying such an expert system, all of the required data would be collected during the routine clinical process step-by-step till the transplantation time.

### Test and evaluation results

The accuracy of the recommendations generated by the program was evaluated based on real patient data and using a confusion matrix. The characteristics of the patient data extracted from paper-based medical records are shown in Table 5. [insert Table 5 here]

**Table 5:** The characteristics of the patient

	Data	Frequency
**Sex**	Female	31 (52.5%)
	Male	28 (47.5%)
**Patient’s status**	Referred	3 (5.1%)
	Waiting list	44 (74.6%)
	Transplanted	7 (11.9%)
	Preassessment	3 (5.1%)
	Referred to committee	2 (3.4%)
**Blood group**	O+	19 (32.2%)
	O-	5 (8.5%)
	A+	5 (8.5%)
	A-	2 (3.4%)
	B+	10 (16.9%)
	B-	3 (5.1%)
	AB+	10 (16.9%)
	AB-	5 (8.5%)

Since the primary goal of this expert system was to identify the most suitable transplant candidate, this evaluation was performed based on the end of decision point 3. The accuracy, sensitivity, and specificity measures of our application according to the confusion matrix (Table 6) were 84.75% (73.01–92.78%; 95% CI), 75.68% (58.80–88.23%;95% CI), and 100%, respectively. Those measures were based on data collected from fifty-nine real case patients who were referred to the LTx clinic (Table 6). [insert Table 6 here]

**Table 6:** Confusion matrix of evaluation

Application Real condition (Actual)	The answer: he/she is to be a candidate (Test positive)	The answer: he/she cannot be a candidate (Test negative)	Total
**The patient could be an eligible LTx candidate**	28 (TP)	9 (FN)	37
**The patient is not an eligible LTx candidate**	0 (FP)	22 (TN)	22
**Total**	28	31	59

†True positive (TP), False negative (FN), False positive (FP), True negative (TN)

Mean scores and sample comments from the heuristic evaluation were organized into Nielsen’s 10 usability heuristics (Table A-2, Appendix A). The score for each question ranges from 0 to five. The zero scores indicated the best performance and five indicated the worst performance.

Screen navigation, language, color, and typographical errors, both in tablet form and smartphone were rechecked by users. Based on users’ views on prototype versions of the system, additional sections were considered in addition to the main sections related to patient management and evaluation. According to users’ opinions, a section was considered an informative section of the system, including the text of the protocols embedded in the expert system and contents related to drug information concerning lung transplantation.

For usability evaluation, six clinicians who work at Imam Khomeini lung transplant center completed the validated PSSUQ questionnaire. The mean age of participants was 49.6 years (SD = ±10.2). Most participants were males (80%) and forty% of participants had more than 20 years of work experience in LTx, while 20% of them had less than five years of experience. The participants answered all the questions completely. The mean and median for each of the subclass scores of the questionnaire are reported in Table 7. [insert Table 7 here]

**Table 7:** The results of the usability evaluation

Factors	User1	User2	User3	User4	User5	User 6	Mean
System Usefulness	7	5.83	5.33	6.67	5.5	6.67	6.1667 ± (0.701)
Information quality	7	6	5.5	7	5.33	7	6.305± (0.792)
Interface quality	7	6	5.25	6.5	5.5	7	6.2083± (0.748)
Overall	7	6	4.83	6.5	6.25	6.67	6.2083± (0.757)

*Scores range from 0 to 7. Seven means a high score

The high score was related to user interface quality. A score above six can be considered a “high” level of usability. Overall, the participants rated the usability of our app as being high. This result confirms the ease of use of this system and its usability. On the other hand, clinicians who worked with the system stated that alerts and recommendations represented by the system during the patient follow-up period certainly avoided medical errors and they can evaluate the patients with high confidence.

## Discussion

The LTx application was developed in collaboration with Imam Khomeini Lung transplantation Center based on a complex clinical pathway for candidate management. The main objective of our study was to provide evidence-based information and recommendations to clinicians to support them in a complex decision-making process to manage LTx candidates. To our knowledge, this is the first mobile-based CDSS implemented based on the LTx protocols for candidate management [20, 35, 36]. Accordingly, we revealed that candidate management is one of the neglected areas in lung transplantation concerning the clinical phase and utilization of health-IT solutions [20].

Since CDSS should simulate the clinical decision-making process done by experts, expert guidance was applied at all stages of system development and definition. Furthermore, involving users in the design and development process could improve the quality and usability of the ultimate system [37]. Consequently, forming a multi-disciplinary team of experts as an expert panel of the system is one of the strengths of this study.

Most guideline-based CDDSs focused only on converting instructions into machine-readable systems without considering clinical settings [38–40]. As we represented in Fig. 4, the modeling of embedded knowledge in the system and the decision process were designed according to the clinical information flow of the candidate management to ensure the correct operation of the program. Besides, studies have shown that CDSSs developed in collaboration with clinicians are more applicable than those produced by industry [13, 41].

Studies revealed that integrating standard clinical workflow into CDSS could be an appropriate solution to ensuring the right decisions are made by healthcare providers at the right time in multidisciplinary clinical tasks [21, 42–44]. Though the combination of both the standard protocols and individual patient data based on the decision points in the clinical workflow is a difficult task [45, 46], providing a standard process for evaluating transplant candidates facilitated patient management processes.

The ultimate goal of this study is to support the lung transplant care team with high-quality patient-specific recommendations according to standard protocols. Our CDSS showed promising results (overall accuracy of 84.75%) to identify the most appropriate LTx candidate. The high accuracy of our CDSS in the initial evaluation showed the complete agreement of embedded knowledge in adherence to our clinical guideline. The highly specific result of the evaluation showed the ability of our decision support tool to designate an individual who is not an eligible candidate for lung transplantation. Considering the high risk of this operation, it can be very helpful that this system does not mistakenly recommend a patient as a suitable transplant candidate. Moreover, usability evaluation demonstrated the positive impact of ImamLTx CDSS to enhance physicians’ work in areas of standard documentation of medication, laboratory tests, and clinical patient data. Automatic recording of medication and laboratory tests not only can facilitate documentation but also it can prevent possible errors in clinical data registration.

Evidence shows that sharing decisions among team members is another challenge in lung transplantation systems [21]. By considering the chat section embedded in our app, all of the users were able to communicate with each other. Accordingly, when the new patient was referred to the transplant center, the system was able to send the message to the LTx group automatically. Furthermore, this communication and data sharing followed secure lines to protect patient confidentiality.

The limitations of the present study naturally include evaluation at a single transplant center. Hence, generalizability to other transplantation centers needs further evaluation and validation with larger and different data sets. Since this study was dedicated to the design and initial evaluation of this system, the interventional evaluation could not be done through randomization, which can be the subject of subsequent studies. Another issue is related to the limitation of resources, budget, and technical facilities that are considered in the design of the study. The next issue is the manual data extraction from paper-based records into the web-based application, which could have caused data loss and entry errors. On the other hand, this application could potentially be linked with electronic medical records of lung transplant patients and minimize data entry errors. Another limitation is the lack of examination of the effect of increased adherence to the protocols and transplant outcomes during this study. This case should also be investigated in future studies focusing on metrics such as survival rates, complications, hospitalization time, and transplant rejection. This application can also be extended regarding patient follow-up after transplantation. It is also possible to design a decision-aid tool to enhance patient self-management connected to the proposed CDSS.

## Conclusion

Through a systematic and methodical approach to lung transplant, CDSS was developed based on the clinical protocols to aid the lung transplant care team with suitable patient-specific recommendations and improve physician access to standard medication documentation, laboratory tests, and patient data. Moreover, providing the transplant care team with appropriate recommendations, alerts, statistical reports, and timely access to patient data facilitated the real-time sharing of management decisions in this complex practice setting.

## Electronic supplementary material

Below is the link to the electronic supplementary material.

Supplementary Material 1

## Data Availability

All data generated or analyzed during this study are included in this published article [and its supplementary information files]. The ImamLTx CDSS app that support the findings of this study are available from the corresponding author upon reasonable request. Due to the intellectual property rights of Imam Lung transplant center and protecting patients’ privacy, the application is not available publicly.

## Declarations

**Ethics approval and consent to participate**.

All experiments and methods were performed following relevant guidelines and regulations. Ethical approval for this study was obtained from the Ethical Committee of the Tehran University of Medical Sciences (IR.TUMS.SPH.REC.1397.161). Informed consent was obtained from all participants and/or their legal guardian(s).

**Consent for publication**.

## List of abbreviations

LTx: Lung transplantation
CDSS: Clinical decision support system
DT: Decision tree
