# Supplementary material for: Design and evaluation of a Mobile-Based decision support system to enhance lung transplant candidate assessment and management: knowledge translation integrated with clinical workflow
Source: BMC Med Inform Decis Mak. 2023 Aug 1;23:145. doi: 10.1186/s12911-023-02249-6 (PMC10394935; doi:10.1186/s12911-023-02249-6)
Supplement: Supplementary file 1 — Supplementary Material 1 [file 12911_2023_2249_MOESM1_ESM.docx]

Appendix A:

Table A-1-The results of consensus for demographic and clinical data elements for minimum data set for lung transplantation management

| # | Data sets | Mean | | Std. Deviation | Variance |
| --- | --- | --- | --- | --- | --- |
|  |  | Statistic | Std. Error | Statistic | Statistic |
| 1 | Name | 5.000 | 0.0000 | 0.0000 | 0.0000 |
| 2 | Sex | 5.000 | 0.0000 | 0.0000 | 0.0000 |
| 3 | Age (Birth date) | 5.000 | 0.0000 | 0.0000 | 0.0000 |
| 4 | Height | 5.000 | 0.0000 | 0.0000 | 0.0000 |
| 5 | Blood group | 5.000 | 0.0000 | 0.0000 | 0.0000 |
| 6 | Marital status | 4.571 | 0.2020 | 0.5345 | 0.286 |
| 7 | Education | 4.571 | 0.2020 | 0.5345 | 0.286 |
| 8 | Telephone No | 5.000 | 0.0000 | 0.0000 | 0.0000 |
| 9 | Telephone No of family | 4.857 | 0.1429 | 0.3780 | 0.143 |
| 10 | City | 4.857 | 0.1429 | 0.3780 | 0.143 |
| 11 | National code | 5.000 | 0.0000 | 0.0000 | 0.0000 |
| 12 | Insurance No^†^ | 4.286 | 0.2857 | 0.7559 | 0.571 |
| 13 | Economic status | 4.571 | 0.2020 | 0.5345 | 0.286 |
| 14 | History of chronic disease | 4.857 | 0.1429 | 0.3780 | 0.143 |
| 15 | Transplantation history | 5.000 | 0.0000 | 0.0000 | 0.0000 |
| 16 | Drug allergies | 4.571 | 0.2974 | 0.7876 | 0.619 |
| 17 | History of mental disorders | 4.714 | 0.1844 | 0.4880 | 0.238 |
| 18 | Smoker | 5.000 | 0.0000 | 0.0000 | 0.0000 |
| 19 | Addicted | 5.000 | 0.0000 | 0.0000 | 0.0000 |
| 20 | CBC | 5.000 | 0.0000 | 0.0000 | 0.0000 |
| 21 | Coagulation Profile and inflammation marker | 4.571 | 0.2974 | 0.7876 | 0.619 |
| 22 | Liver and Renal Function | 5.000 | 0.0000 | 0.0000 | 0.0000 |
| 23 | Electrolytes | 4.571 | 0.2974 | 0.7876 | 0.619 |
| 24 | Lipid Profile and blood glucose | 4.857 | 0.1429 | 0.3780 | 0.143 |
| 25 | Thyroid Function | 4.857 | 0.1429 | 0.3780 | 0.143 |
| 26 | Viral Markers | 4.857 | 0.1429 | 0.3780 | 0.143 |
| 27 | ABG | 4.857 | 0.1429 | 0.3780 | 0.143 |
| 28 | PAP | 5.000 | 0.0000 | 0.0000 | 0.0000 |
| 29 | RVEF | 5.000 | 0.0000 | 0.0000 | 0.0000 |
| 30 | LVEF | 5.000 | 0.0000 | 0.0000 | 0.0000 |
| 31 | RV size | 4.857 | 0.1429 | 0.3780 | 0.143 |
| 32 | LV size | 4.571 | 0.2974 | 0.7876 | 0.619 |
| 33 | FEV1 | 5.000 | 0.0000 | 0.0000 | 0.0000 |
| 34 | FVC | 5.000 | 0.0000 | 0.0000 | 0.0000 |
| 35 | FEV1/FVC | 4.857 | 0.1429 | 0.3780 | 0.143 |
| 36 | TLC^†^ | 3.429 | 0.3689 | 0.9759 | 0.952 |
| 37 | RV† | 3.143 | 0.4041 | 1.0690 | 1.143 |
| 38 | RV/TLC^†^ | 3.143 | 0.4041 | 1.0690 | 1.143 |
| 39 | PEF^†^ | 3.143 | 0.4041 | 1.0690 | 1.143 |
| 40 | DLCO | 3.800 | 0.7348 | 1.6432 | 2.700 |
| 41 | DLCO/VC^†^ | 3.143 | 0.4041 | 1.0690 | 1.143 |
| 42 | 6MWT info | 4.174 | 0.1844 | 0.4880 | 0.238 |
| 43 | Type of visit | 5.000 | 0.0000 | 0.0000 | 0.0000 |
| 44 | Data of visit | 4.714 | 0.1844 | 0.4880 | 0.238 |
| 45 | SpO2 | 4.857 | 0.1429 | 0.3780 | 0.143 |
| 46 | HR | 4.00 | 0.4880 | 1.291 | 2.667 |
| 47 | RR^†^ | 3.200 | 0.5831 | 1.3038 | 1.700 |
| 48 | blood pressure | 4.571 | 0.2974 | 0.7876 | 0.619 |
| 49 | Weight (kg) | 4.143 | 0.4592 | 1.2150 | 1.476 |
| 50 | C.C | 4.714 | 0.1844 | 0.4880 | 0.238 |
| 51 | P.I | 4.714 | 0.1844 | 0.4880 | 0.238 |
| 52 | Assessment and plan^†^ | 3.200 | 0.5831 | 1.3038 | 1.700 |
| 53 | Drug name | 4.857 | 0.1429 | 0.3780 | 0.143 |
| 54 | Dosage | 4.857 | 0.1429 | 0.3780 | 0.143 |
| 55 | Prescription | 4.571 | 0.2974 | 0.7876 | 0.619 |
| 56 | Date of prescription | 4.571 | 0.2974 | 0.7876 | 0.619 |
| 57 | DX | 5.000 | 0.0000 | 0.0000 | 0.0000 |
| 58 | Referral data | 5.000 | 0.0000 | 0.0000 | 0.0000 |
| 59 | LTx status | 5.000 | 0.0000 | 0.0000 | 0.0000 |
| 60 | Living status | 4.571 | 0.2974 | 0.7876 | 0.619 |
| 61 | Committee data | 4.714 | 0.1844 | 0.4880 | 0.238 |
| 62 | LTx date | 5.000 | 0.0000 | 0.0000 | 0.0000 |
| 63 | LTx type | 5.000 | 0.0000 | 0.0000 | 0.0000 |
| † These data sets excluded because they didn't reach expert consensus. | | | | | |

Table A-2- The Nielsen usability scores

| Nielsen’s 10 usability heuristics | Mean (SD) |
| --- | --- |
| Visibility of system status | 0.33± (0.516) |
| Match between system and the real world | 0.5± (0.547) |
| User control and freedom | 0.5 ± (0.547) |
| Consistency and standards | 0.166 ± (0.408) |
| Help users recognize, diagnose, and recover from errors | 0.33 ± (0.516) |
| Error prevention | 0.667 ± (0.816) |
| Recognition rather than recall | 0.666 ± (0.516) |
| Flexibility and efficiency of use | 0.334 ± (0.516) |
| Esthetic and minimalist design | 0.5 ± (0.547) |
| Help and documentation | 0 |


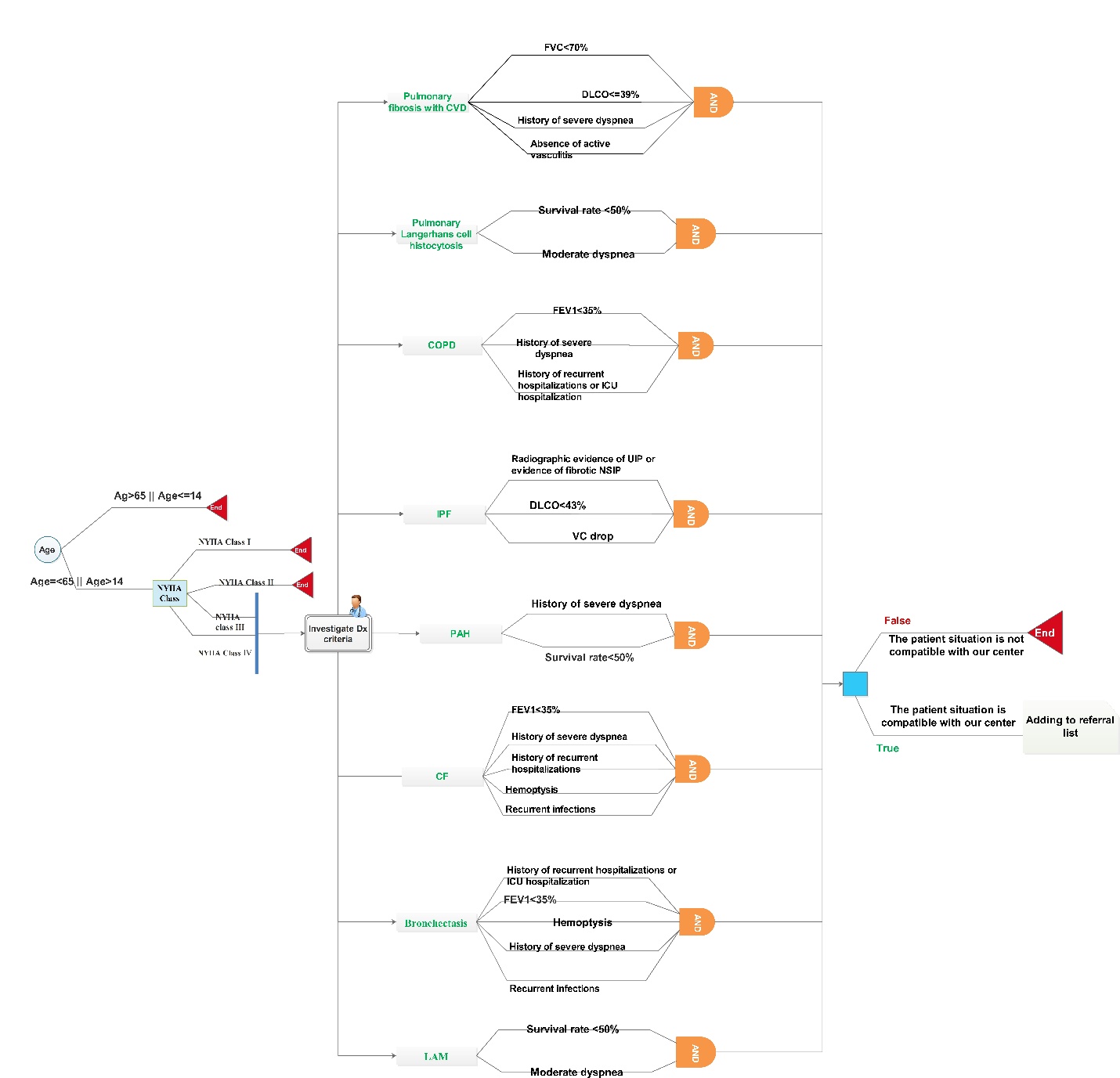


Fig A-1. The sample of decision models for candidate evaluation based on disease diagnosis


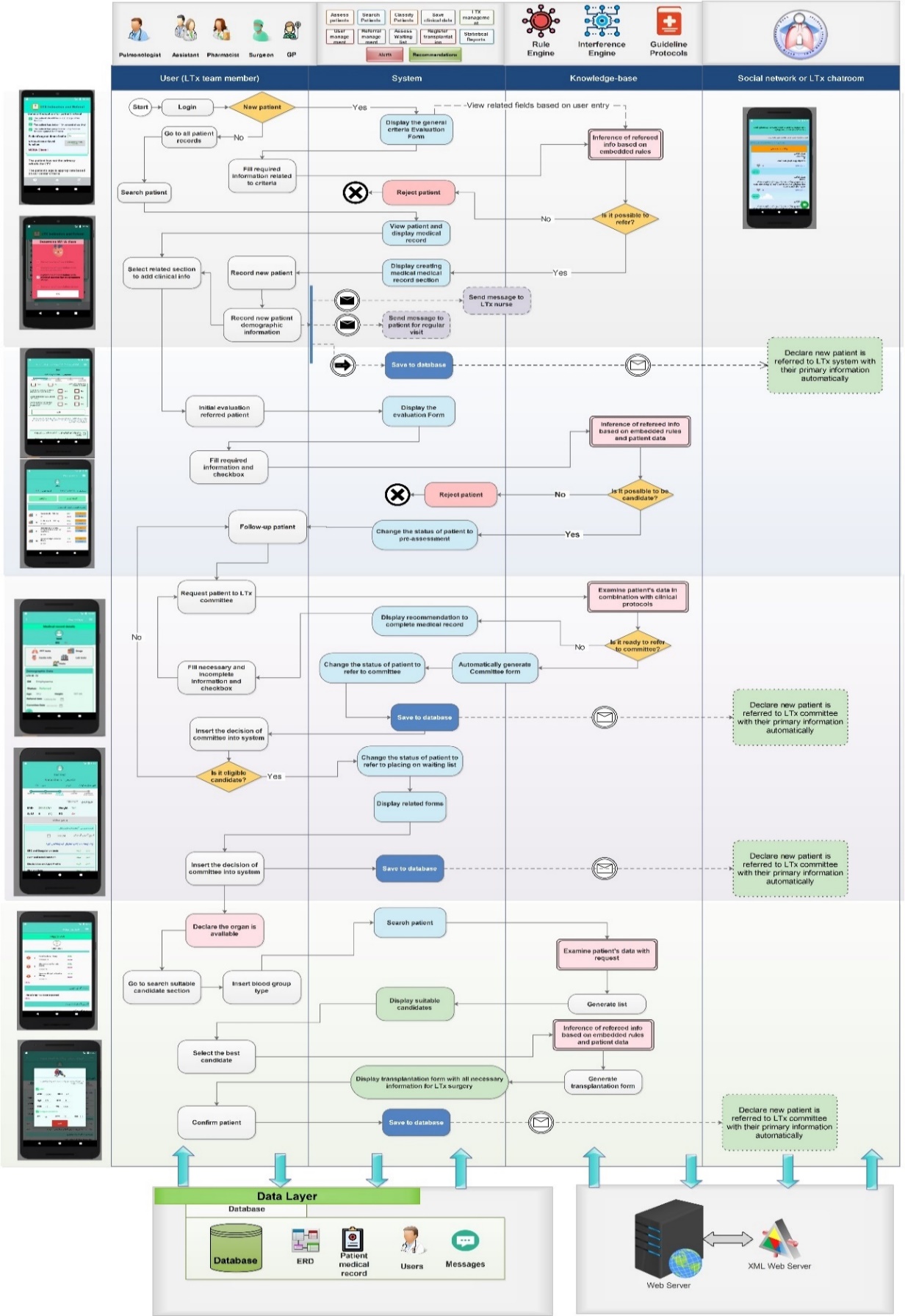


Fig A-2. The functional model of the ImamLTx app

| 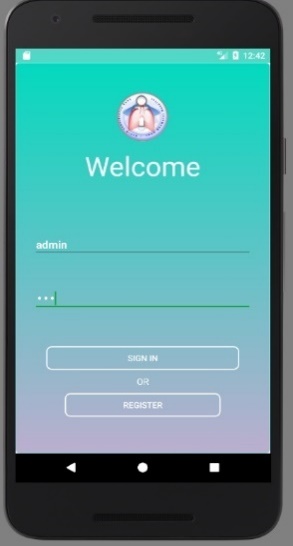  a | 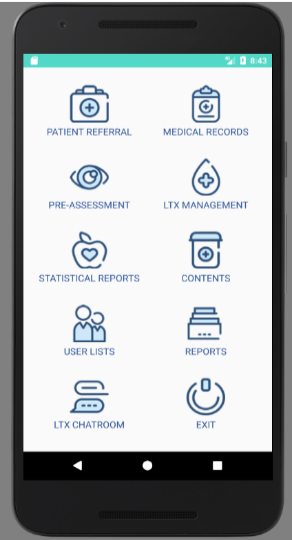  b | 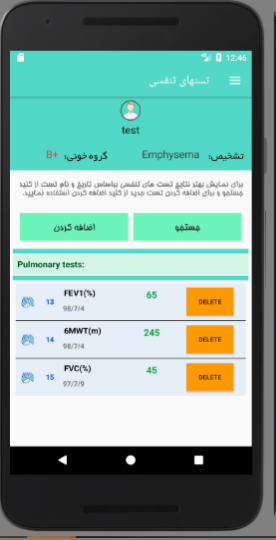  c | 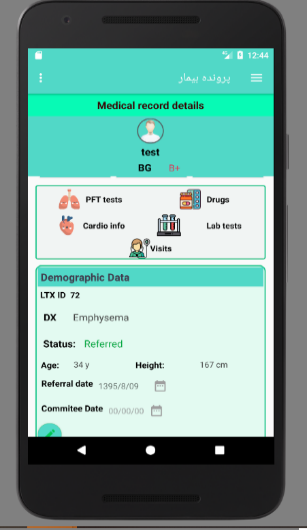  d |
| --- | --- | --- | --- |
| 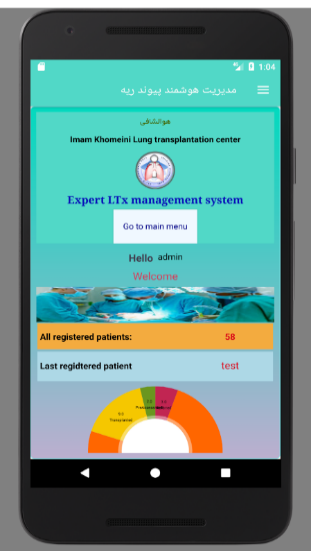  e | 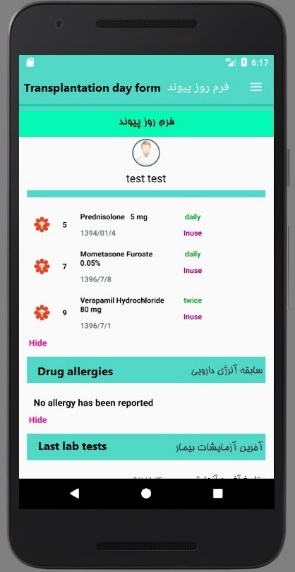  f | 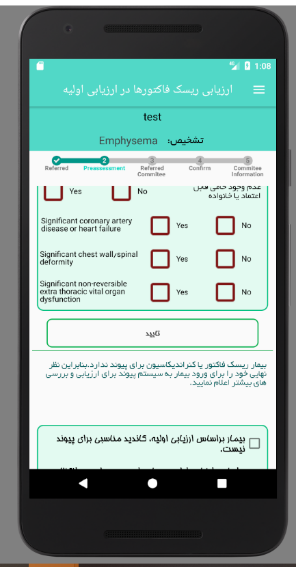  g | 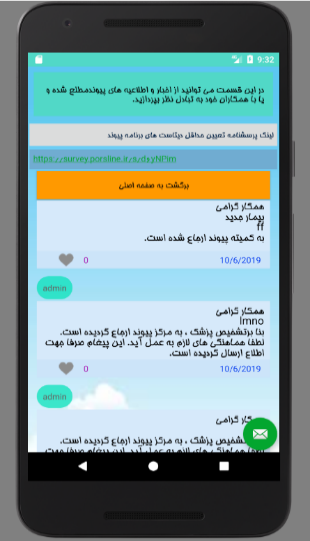  h |

Fig A-3- Some images of ImamLTx CDSS. (a. Login page, b. Main menu, c. Add pulmonary function test results, d. The patient medical record for each patient, e. Statistical reports, f. Generating whole reports of patient data automatically, g. Initial assessment of transplant candidate, h. chatroom)
